# Assessment of critical steps of a GC/MS based indirect analytical method for the determination of fatty acid esters of monochloropropanediols (MCPDEs) and of glycidol (GEs)

**DOI:** 10.1016/j.foodcont.2017.01.024

**Published:** 2017-07

**Authors:** Zuzana Zelinkova, Anupam Giri, Thomas Wenzl

**Affiliations:** European Commission, Joint Research Centre, Retieseweg 111, B-2440 Geel, Belgium

**Keywords:** 3-MCPD esters, 2-MCPD esters, Glycidyl esters, Food contaminants, Indirect analysis, GC-MS, Food

## Abstract

Fatty acid esters of 2- and 3-chloropropanediol (MCPDEs) and fatty acid esters of glycidol (GEs) are commonly monitored in edible fats and oils. A recommendation issued by the European Commission emphasizes the need of generating data on the occurrence of these substances in a broad range of different foods. So far, analytical methods for the determination of MCPDEs and GEs are fully validated only for oils, fats and margarine. This manuscript presents the assessment of critical steps in the AOCS Cd 29a-13 method for the simultaneous determination of MCPDEs and GEs in the fat phase obtained from bakery and potato products, smoked and fried fish and meat, and other cereal products. The trueness of the method is affected by the additional formation of 3-MBPD esters from monoacylglycerols (MAGs), which are frequently present in food. The overestimation of GE contents for some samples was confirmed by the comparison of results with results obtained by an independent analytical method (direct analysis of GE by HPLC-MS/MS). An additional sample pre-treatment by SPE was introduced to remove MAGs from fat prior to the GEs conversion, while the overall method sensitivity was not significantly affected. Trueness of the determination of GEs by the modified analytical procedure was confirmed by comparison with a direct analysis of GEs. The potential impact on accuracy of results of the final sample preparation step of the analytical procedure, the derivatization of free forms MCPD and MBPD with PBA, was evaluated as well. Different commercial batches of PBA showed differences in solubility in a non-polar organic solvent. The PBA derivatization in organic solvent did not affect precision and trueness of the method due to the isotopic standard dilution. However, method sensitivity might be significantly compromised.

## Highlights

• Esters of monochloropropanediols and glycidyl esters are food contaminants generated during food processing.
• Extension of an analytical method standardised for oils and fats to other food matrices is critically evaluated.
• Trueness of the method was assessed by comparision with other independent method.
• Presence of monoacylglycerols in food sample caused biased results of glycidyl esters.
• Additional SPE clean-up is introduce to accurate analytical results.

## 1 Introduction

Fatty acid esters of 2-/3-chloropropane-1,2-diol (2-/3-MCPDEs) and of glycidol (GEs) might be generated during food processing (Hamlet et al., 2011, Hrncirik and Duijn, 2011). The presence of chlorinated propanols, particularly 3-MCPD, in food is well known since 1970's when this substance was discovered by Velisek et al. (1978) in acid-hydrolysed vegetable proteins (acid-HVP). Esters of MCPD were also found in acid-HVP (Davidek, Velisek, Kubelka, Janicek, & Simicova, 1980), but the majority of investigations has started quite recently after reporting high levels in foods and in particular in refined edible oils (Weisshaar, 2008, Zelinkova et al., 2006). GEs have been detected in the frame of MCPDEs analysis in vegetable oils (Weisshaar & Pere, 2010). Free forms of these substances (3-MCPD and glycidol) released from their esterified forms during digestion have been classified as carcinogenic to humans (group 2B and 2A, respectively) (IARC, 2000, IARC, 2012).

Preliminary exposure assessment of the European Food Safety Authority (EFSA) on 3-MCPD in food identified margarine and vegetable fats and oils as major contributors to dietary exposure, followed by bread and fine bakery wares (EFSA, 2013). EFSA concluded that for 3-MCPD a tolerable daily intake (TDI) of 0.8 μg/kg body weight is appropriate, whereas a margin of exposure of 25000 was considered of low health concern in case of glycidol (EFSA, 2016).

The European Commission issued in 2014 Commission Recommendation 2014/661/EU on the monitoring of free MCPD, MCPDEs and GEs in food. MCPDEs and GEs were recommended to be monitored in several food groups, comprising fine bakery ware, bread and rolls, smoked meat and fish, potato- and cereal-based snacks, fried potato products and vegetable oil containing foods. Analytical methods standardised by the American Oil Chemists Society (AOCS) were suggested to be used as basis for analysis, but these methods covered only edible oils and fats (EC, 2014).

In general, analytical methods for the determination of MCPDEs and GEs follow two distinct routes. The direct analysis of fatty acid esters by HPLC-MS comprises one possibility, which however entails the measurement of a large number of substances (individual fatty acid esters of MCPD/glycidol) (Haines et al., 2011, Hori et al., 2012). The strong similarity of target compounds with major matrix constituents (in particular mono- and diacylglycerols) hampers the separation of MCPDEs and GEs from the oil matrix. However, satisfactory separation can be achieved for GEs by applying SPE or gel permeation chromatography (GPC) clean-up (Dubois et al., 2011, Musukawa et al., 2011). Consequently, a direct analytical method for the determination of GEs in fats and oils was standardised by AOCS (AOCS/JOCS, 2012). The direct analysis of MCPDEs on routine basis is so far hardly applied and analytical methods for the direct determination of MCPDEs in whatever food have not been fully validated yet.

The second route consists of the indirect determination of MCPDEs and GEs via the MCPD/glycidol moieties. The analytical methods entail the cleavage of MCPD/glycidol from its esterified form, and determination of the total amount of the so called bound MCPD/glycidol (Divinova et al., 2004, Ermacora and Hrncirik, 2013, Kuhlmann, 2011, Küsters et al., 2010, Weisshaar, 2008). Several methods have been developed for the indirect analysis of MCPDEs, however all are following a similar protocol (cleavage of MCPD, clean-up, derivatization, GC/MS analysis). The important two steps are the cleavage of MCPD from their esterified form (transesterification) and derivatization prior to GC/MS analysis. Both of these steps have been already well optimized and the performance of analytical methods was evaluated by collaborative studies (Fry et al., 2013, Karasek et al., 2013). The cleavage of MCPD (transesterification) is carried out under acidic or alkaline conditions in the presence of methanol to form fatty acids methyl esters and MCPD. Due to the low volatility and high polarity of MCPD, derivatization prior to the GC/MS analysis is necessary.

Relatively new is the methodology for indirect GE determination. The determination of GEs has been incorporated into the existing indirect methods for MCPDE determination and follows the same analytical procedure. This was achieved by conversion and thereby stabilisation of GEs to either a compound structurally similar to MCPD - bromopropandiol (MPBD) or to MCPD itself (DGF, 2011, Ermacora and Hrncirik, 2013, Kuhlmann, 2011). In the first case the phenylboronic acid (PBA) derivative of 3-MBPD is determined by GC/MS as an equivalent of glycidol, in the second case the PBA derivative of MCPD is determined as a sum of bound MCPD and bound glycidol. Indirect methods were considered more suitable for routine application.

A number of analytical methods had been standardised for the indirect analysis of MCPDEs and GEs (AOCS, 2013a, AOCS, 2013b, AOCS, 2013c) in fats and oils and oil-based emulsions (AOCS, 2015). A standardised method for the determination of MCPDEs and GEs in foods other than fats and oils does not exist yet. Our group has investigated the performance of the AOCS methods mentioned in the Commission Recommendation for foods other than fats and oils, considering the broad scope of the monitoring plan issued by the European Commission and consequently the variety of matrices that has to be dealt with. AOCS Cd 29a-13 was selected, as this method allows the determination of MCPDE and GE content within a single assay (AOCS, 2013a). The analytical procedure consists of the conversion of GEs to MBPDEs, followed by acid catalysed transesterification and cleavage of MCPDEs and MBPDEs. The released free forms of MCPD and MBPD are further derivatized with PBA and determined by GC/MS.

For certain groups of foods, in particular those containing partial glycerides used as emulsifiers we observed somewhat elevated levels of GEs and speculated that reactions carried out in the course of sample preparation may affect the trueness of the method as artefact formation might occur (additional formation of MBPD, transformation of GEs into MCPDEs and vice versa). Ermacora and Hrncirik (2013) already reported an unfavourable influence of partial glycerides on the artefact formation of MBPD.

The aim of the presented study was to critically evaluate the applicability of the selected method to the fat phase obtained from different food matrices. Several aspects were considered including the impact of sample composition and content of potential precursors on the accuracy of the analytical results. Main focus was given to the artefact free conversion of GEs into 3-MBPDEs, which was identified as a critical step having a potential impact on the trueness of the method. The final sample preparation step, the derivatization with PBA, and the influence of the particular batch of commercial derivatization reagent were evaluated as well.

## 2 Materials and methods

### 2.1 Food samples

A set of 12 food samples representing different food categories was purchased in Belgian retail markets. Extra virgin olive oil, used as a blank sample, was obtained from a local producer in Greece and palm oil from the European Federation of the Oil and Proteinmeal Industry (FEDIOL). A spiked soybean oil was used for analytical quality control purposes. It contained the following analyte amounts, expressed as equivalents to the free forms: 3-MCPD 2.88 ± 0.29 mg/kg; 2-MCPD < 0.10 mg/kg; glycidol 4.25 ± 0.68 mg/kg (glycidyl laurate 2.01 mg/kg; glycidyl palmitate 5.74 mg/kg; glycidyl stearate 1.53 mg/kg; glycidyl oleate 6.56 mg/kg, glycidyl linoleate 0.49 mg/kg; glycidyl linolenate 1.39 mg/kg). All samples were homogenized and kept according to the labelled storage recommendations.

### 2.2 Reagents and materials

All solvents were of at least analytical grade, purchased from either Sigma-Aldrich (Diegem, Belgium) or VWR (Leuven, Belgium). Sodium polyacrylate cross-linked, sand 50–70 mesh particle size, sulphuric acid (≥95%), sodium hydrogen carbonate, anhydrous sodium sulphate and sodium bromide were obtained from Sigma-Aldrich (Diegem, Belgium). Aminopropyl (NH_2_) SPE cartridges (Extract Clean™, 500 mg, 4.0 mL) and HPLC syringe filters (regenerated cellulose, 13 mm, 0.2 μm) were purchased from Grace Davison Discovery Science (Deerfield, IL, USA). Four different batches of phenylboronic acid (PBA) reagent were obtained for comparison purposes from different suppliers, three from Sigma-Aldrich (Diegem, Belgium) and one from ACROS (Geel, Belgium).

The standard compounds 1,2-dipalmitoyl-3-chloropropanediol (diP-3-MCPD, CAS#51930-97-3); 1,3-distearoyl-2-chloropropanediol (diS-2-MCPD, CAS#26787-56-4); glycidyl laurate (GE-L, CAS#1984-77-6); glycidyl palmitate (GE-P, CAS#7501-44-2); glycidyl stearate (GE-S, CAS#7460-84-6) as well as the isotopically labelled compounds 1,2-dipalmitoyl-3-chloropropanediol-d_5_ (diP-3-MCPD-d_5_); 1,3-distearoyl-2-chloropropanediol-d_5_ (diS-2-MCPD-d_5_) and glycidyl oleate-d_5_ (GE-O-d_5_) were obtained from Toronto Research Chemicals Inc. (Toronto, Canada). Standard solutions of glycidyl oleate (GE-O, CAS#5431-33-4); glycidyl linoleate (GE-Li, CAS#24305-63-3); glycidyl linolenate (GE-Ln, CAS#51554-07-5) and glycidyl palmitate-d_5_ (GE-P-d_5_) in toluene were purchased from Chiron AS (Trondheim, Norway). Certified standard of a mixture of 1-monooleoyl-rac-glycerol (CAS#111-03-5), 1,2-dioleoyl-rac-glycerol (CAS#3738-74-7), 1,3-diolein (CAS#2465-32-9) and triolein (CAS#122-32-7) was received from Supelco Analytical (Bellefonte, PA, USA). 1,2-Dipalmitoyl-rac-glycerol (CAS#40290-32-2) and α-monopalmitin (CAS#542-44-9) were obtained from Sigma-Aldrich.

Stock solutions of individual compounds were prepared gravimetrically in toluene and methanol for the indirect determination of MCPDEs and GEs, and the direct determination of GEs, respectively. Calibration standard solutions were made by dilution in toluene (diP-3-MCPD, diS-2-MCPD, GE-P; 0.02–2.5 μg/mL equivalent to free form) and dilution in methanol:2-propanol (1:1, v/v; GE-L, GE-P, GE-S, GE-O, GE-Li, GE-Ln; 5–200 ng/mL) for indirect and direct analysis, respectively. Calibration standards of mono- and diolein were prepared by dilution in 2,2,4-trimethylpentane (10–500 μg/mL). All standard solutions were kept at 5 °C.

### 2.3 Equipment

A pressurised liquid extractor ASE 300 (Dionex, Sunnyvale, CA, USA) was used for the extraction of the samples. Sample extracts were evaporated on a Turbo Vap^®^ workstation (Zymark, Hopkinton, MA, USA) respectively on a Techne Sample Concentrator Dri-Block^®^ DB-3D (Bibby Scientific, Staffordshire, UK). GPC was performed on a column (450 mm length, 10 mm id) filled with Bio-Beads^®^ S-X3, obtained from Bio-Rad Laboratories (Hercules, CA, USA). The GPC column was connected to an Agilent 1100 series HPLC system (Agilent Technologies, Santa Clara, CA, USA) consisting of a binary pump (G1312A), degasser (G1379A), autosampler (G1329A), diode array detector (G1315B) and fraction collector (G1364C).

Mono- and diacylglycerol content was determined on an Agilent 1100 series HPLC system comprising a quaternary pump (G1311A), degasser (G1322A), autosampler (G1329A), column compartment equipped with an Ascentis^®^ Si column (10 cm × 2.1 mm, 3 μm; Supelco Analytical) and connected to an Alltech 3300 evaporative light scattering detector (ELSD, Grace Davison Discovery Science).

A gas chromatograph (GC) 6890N (Agilent Technologies, Santa Clara, CA, USA) with split/splitless injector equipped with a J&W DB-5 MS GC column (30 m × 250 μm × 0.25 μm, Agilent Technologies) was used for the indirect analysis of MCPDEs and GEs. The GC was coupled to an Agilent 5973 inert single quadrupole mass spectrometer (Agilent Technologies) operated in electron ionisation (EI) mode at 70 eV. Injection was carried out using an automated Gerstel MPS injection system (Gerstel, Mulheim and der Ruhr, Germany). Recorded data were evaluated by MSD ChemStation E.02.00.493 (Agilent Technologies).

For the direct determination of GEs, a HPLC system Nexera X2 (Shimadzu, 's-Hertogenbosch, The Netherlands), consisting of two pumps (LC-30AD), degasser (DGU-20A), autosampler (SIL-30AC) and column oven (CTO-30A) equipped with an Eclipse XDB-C18 column (15 cm × 4.6 mm, 5 μm; Agilent Technologies), hyphenated to a hybrid quadrupole linear ion trap mass spectrometer QTRAP^®^ 6500 (AB SCIEX, Darmstadt, Germany) was used. The mass spectrometer was operated in positive atmospheric pressure chemical ionisation mode. Experiments were performed in multiple reactions monitoring mode (MRM). Acquired data were evaluated by means of MultiQuant™ 3.0.2 software (AB SCIEX).

### 2.4 Fat extraction

The analytical method reported by Wenzl et al. (2015) and Samaras et al. (2016) was followed for the extraction of fat from food. Briefly 5 g of sample were mixed with 5 g sand and 15 g sodium polyacrylate and transferred into 33 mL ASE extraction cell. The sample amount was increased to 15–20 g in case of food with low fat, which were placed into 100 mL ASE cell with proportionally increased amount of sand and sodium polyacrylate. The extraction was carried out with *tert*-butyl methyl ether (TBME) at 40 °C in 2 cycles of 5 min static time with 60% purge volume and 180 s purge time. Extract were evaporated until dryness in a Turbo Vap^®^ workstation at 40 °C using a steam of nitrogen gas (N_2_). The fat content was determined gravimetrically for each test material in triplicate.

### 2.5 MCPDEs and GEs determination – indirect method

The determination of MCPDEs and GEs was carried out as described in AOCS standard method Cd 29a-13 (AOCS, 2013a). A portion of 100 mg of the extracted fat was weighed into a 10 mL glass tube, spiked with 100 μL mixed internal standard solution (containing diP-3-MCPD-d_5_, diS-2-MCPD-d_5_, GE-O-d_5_, each 2.5 μg/mL corresponding to the free forms of MCPD and glycidol) and dissolved in 2 mL of tetrahydrofuran (THF). Conversion of GEs to MBPDEs was done by applying 30 μL of NaBr acid aqueous solution (3.3 mg/mL, 5% H_2_SO_4_) and incubating at 50 °C for 15 min. The reaction was stopped (3 mL 0.6% NaHCO_3_, v/v) and the target compounds were extracted with 2 mL *n*-heptane. The extract was evaporated at 40 °C with a stream of N_2_ and the residue was dissolved in 1 mL THF. Transesterification was performed for 16 h at 40 °C after adding to the solution in THF 1.8 mL sulphuric acid solution in methanol (1.8%, v/v). The reaction was stopped (0.5 mL 9% NaHCO_3_, v/v) and the organic solvents were evaporated at 40 °C with a stream of N_2_. Fatty acid methyl esters were separated from the sample by liquid-liquid extraction (2 mL 20% Na_2_SO_4_, w/v; 2 × 2 mL *n*-heptane). Derivatization was carried out in an ultrasonic bath at room temperature for 5 min with 200 μL PBA solution (250 mg/mL, acetone:H_2_O 19:1, v/v). The phenylboronate derivatives were extracted with *n*-heptane (2 × 1 mL), evaporated at 40 °C with a stream of N_2_ and re-dissolved in 300 μL 2,2,4-trimethylpentane. One microliter was injected into the GC/MS. The whole procedure was done in triplicate.

It has to be highlighted that the GC/MS instrument measures PBA derivatives of 3-MBPD and 2- and 3-MCPD. However, all results were expressed for GEs (3-MBPD) in equimolar amounts of glycidol (glycidol equivalents) and for 2-/3-MCPDEs in equimolar amounts of 2- and 3-MCPD. Further on the terms measured/determined glycidol contents and 2-/3- MCPD contents, respectively, in place of PBA derivatives of the respective analytes is used in the text.

#### 2.5.1 GC/MS analysis

For the GC/MS analysis, 1 μL of sample extract was injected in pulsed splitless mode (pulse pressure 200 kPa for 0.30 min) at a temperature of 250 °C into the split/splitless injector. Helium was used as carrier gas at a flow rate of 1.2 mL/min. The transfer line temperature was set to 300 °C. The oven temperature program was as follows: initial temperature 60 °C held for 1 min, 6 °C/min till 150 °C (held for 2 min), 30 °C/min till 300 °C held for 10 min. The target analytes were detected in selected ion monitoring mode (SIM). Detection parameters are listed in Table 1.

#### 2.5.2 SPE clean-up

Approximately 100 mg fat was weighted into a 1.5 mL vial, spiked with 100 μL of mixed internal standard solution (diP-3-MCPD-d_5_, diS-2-MCPD-d_5_, GE-O-d_5_, 2.5 μg/mL recalculated to respective free form) and dissolved in 500 μL *n*-hexane:ethyl acetate (85:15, v/v). The SPE cartridge was conditioned with 2 mL elution solvent (*n*-hexane:ethyl acetate, 85:15, v/v). The sample was loaded on the cartridge and target compounds were eluted with 10 mL elution solvent. The collected fraction was evaporated at 40 °C under a stream of nitrogen. The obtained residue was dissolved in 2 mL tetrahydrofuran and used for the indirect determination of MCPDEs and GEs starting directly with the conversion of GEs to MBPDEs.

### 2.6 GEs determination – direct method

100 mg of fat was weighed into a 1.5 mL vial, followed by addition of 100 μL internal standard (GE-P-d_5_, 2.0 μg/mL) and dissolved in 1 mL cyclohexane:ethyl acetate (1:1, v/v). The sample was homogenized, 1 mL was injected onto the GPC column and eluted with cyclohexane:ethyl acetate (1:1, v/v) at a flow rate of 1 ml/min. The GE containing fraction was collected between 22 min and 42 min of the 45 min total run time. The collected fraction was evaporated until dryness at 40 °C with a stream of N_2_, dissolved in 1 mL methanol:2-propanol (1:1, v/v), filtered through a regenerated cellulose filter and transferred into a 1.5 mL autosampler vial for HPLC-MS/MS analysis. The full analysis was done in triplicate.

#### 2.6.1 HPLC-MS/MS analysis

HPLC conditions were adopted from AOCS standard method Cd 28-10 (AOCS/JOCS, 2012). The mobile phase A (methanol:H_2_O, 92:8, v/v) and mobile phase B (2-propanol) were programmed as follows: isocratic elution 100% mobile phase A till 18.0 min, linear gradient 100% B till 18.1 min, isocratic elution 100% B till 25.0 min, linear gradient 100% A till 25.1 min, isocratic elution 100% A till 35.0 min. The flow rate was 1 mL/min, the injection volume was 20 μL, the column temperature was maintained at 40 °C. Atmospheric pressure chemical ionisation was performed at ion source gas pressure of 30 psi, vaporization temperature of 500 °C, needle current of 2.5 μA, entrance potential of 10.0 V and curtain gas pressure of 25.0 psi. Collision cell parameters and monitored ion transitions are compiled in Table 2.

### 2.7 Partial acylglycerols determination

The extracted fat was weighed (100 mg) into a glass vial and dissolved in 5 mL 2,2,4-trimethylpentane. A mixture of 2,2,4-trimethylpentane:tetrahydrofuran (1:3, v/v) was used in case the fat was not fully soluble in 2,2,4-trimethylpentane. About 1 mL of the sample was filtered through a regenerated cellulose filter and an aliquot of 500 μL was diluted with 500 μL 2,2,4-trimethylpentane and subjected to HPLC/ELSD analysis. The analysis was done in duplicate for each sample.

#### 2.7.1 HPLC-ELSD analysis

Separation of lipid classes was accomplished according to the HPLC method published by Torres, Vazquez, Senorans, and Reglero (2005). Three mobile phases were used (A = 2,2,4-trimethylpentane, B = 2-propanol, C = *tert*-butyl methyl ether) with a linear gradient which is given in the following: initial conditions 99.5% A and 0.5% C for 1 min, gradient to 78% A and 22% C till 15 min, gradient to 70% A, 5% B and 25% C till 30 min, gradient to 69% A, 6% B and 25% C till 35 min, gradient to 99.5% A, 0.5% C till 36 min, isocratic column conditioning at 99.5% A, 0.5% C till 45 min. The mobile phase flow rate was set to 0.4 mL/min and 3 μL sample was injected. The ELSD was maintained at 40 °C, with a N_2_ flow rate of 1.5 mL/min and a gain factor of 16.

### 2.8 Statistical software

Design of experiment was performed with statistical software R, version 3.0.2. for Windows (http://www.r-project.org). Multiple range tests were applied to determine significant difference between values using Statgraphics, version 15.2.06 (StatPoint, Inc.).

## 3 Results and discussion

The epoxide group of GEs is highly reactive under acidic conditions. It can react with a variety of nucleophiles (water, alcohols, thiols, amines, acids, halides etc.) (Bronsted & Kilpatrick, 1959). To prevent uncontrolled reactions and to increase chemical stability of the analytes, GEs are transformed into more stable MBPDEs. An acidified aqueous solution of sodium bromide is used for the epoxide ring opening and conversion of GE to MBPDEs. Further the MBPDEs are subjected to the same chemical reactions (transesterification, derivatization) as applied for MCPDEs. This means GEs are subjected to three chemical reactions for converting them into a compound suitable for GC/MS detection. Considering the laborious procedure and many possibilities for bias, the performance of the method has to be evaluated carefully. So far a lot of effort was spent on the optimization of the method for the determination of MCPDEs and GEs in edible oils and fats. Particularly the reaction conditions of the conversion of GEs to MBPDEs and the influence of the composition of the edible oil on the analytical results were studied (Ermacora & Hrncirik, 2013). The particular analytical method was validated by collaborative study and accepted as official method by AOCS (2013a). The extension of this method to food other than edible fats and oils, as recommended by EC, required the in depth study of method performance, as chemical reactions potentially occurring during sample preparation might lead to biased results. Thus, trueness of the method had to be assessed for each food category.

Several food samples, representing different food groups listed in the Commission Recommendation (EC, 2014) were selected for method validation. Fat was extracted and gravimetrically determined. MCPDEs and GEs were measured in the extracted fat according to AOCS method Cd 29a-13 (Table 3). Limit of quantification of the method was below 0.02 mg/kg, indicated by a signal-to-noise ratio higher than 10. Repeatability, determined at three concentration levels (0.05; 0.5; 2.5 mg/kg, spiked extra virgin blank oil with standards of 2-/3-MCPDEs and GEs), was within the range of 0.7–8.2% for 3-MCPD, 1.4–5.1% for 2-MCPD and 2.1–6.9% for glycidol. The performance of the method was monitored by including the QC soybean oil in each sample batch.

### 3.1 Experimental design

**Fig. 1:**
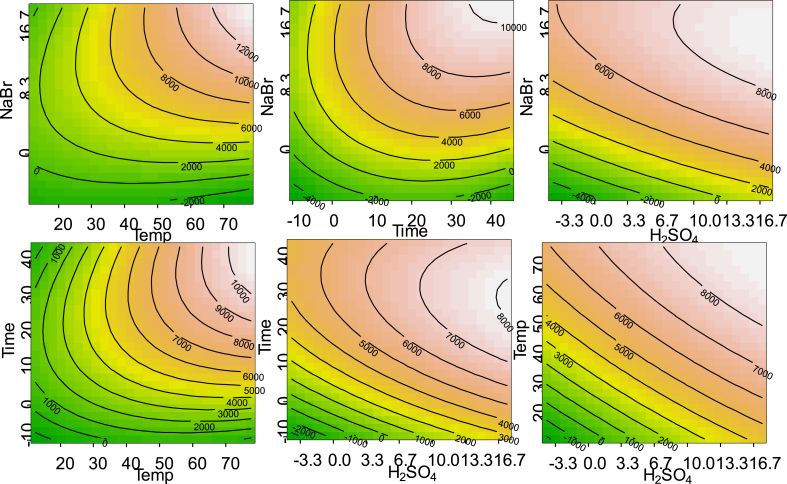
Contour plot for the response for 3-MBPD as a function of NaBr content, H_2_SO_4_ concentration, time and temperature.

A critical step of the analysis is the conversion of GEs to 3-MBPDEs. Design of experiment was applied to explore the influence of reaction conditions on 3-MBPDEs and to optimize reaction parameters. The reaction was described as a function of parameters such as amount of NaBr, H_2_SO_4_ concentration, reaction time and reaction temperature and was modelled by the use of response surface methodology for the contaminated palm oil. A central composite design was chosen to show the effects of selected parameters on the reaction efficiency. The range of the variables was defined considering the previously optimized conditions for oils and fats as specified in the AOCS official method. The following range for the variables was applied: 0–20.8 mg/mL NaBr, 0–18.3% H_2_SO_4_, 0–45 min reaction time and 5–80 °C reaction temperature. Measured abundance for 3-MBPD was normalized by the amount of sample intake and results were visualised by response contour plots (Fig. 1). Each contour represents the effect of two variables on the response of 3-MBPD. The graphs demonstrate that the response did not have maxima within the selected parameter range. They all show a continuous increase of the response with increasing variable levels. Moreover the response plots indicate that the amount of NaBr has the main effect on the 3-MBPD response, whereas the concentration of H_2_SO_4_ was less important.

**Fig. 2:**
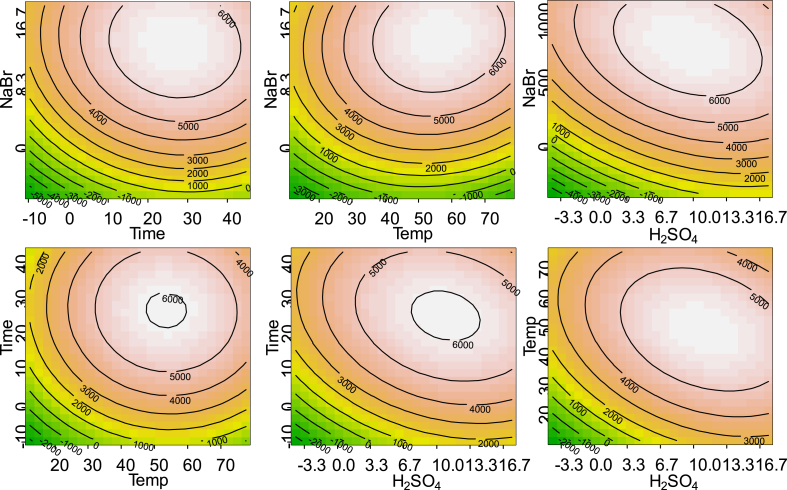
Contour plots for the response of stable isotope labelled 3-MBPD-d_5_ as a function of NaBr content, H_2_SO_4_ concentration, time and temperature.

The stable isotope labelled internal standard GE-O-d_5_, which was added to the test portion prior to sample preparation, was utilized to elucidate whether the lack of maxima in the response plots for 3-MBPD were caused simply by improper selection of the variable range, or if artefact formation occurred. Fig. 2 presents, for the same variable combinations as shown in Fig. 1, 2D contour plots for the response obtained for 3-MBPD-d_5_. Each plot shows a maximum within the experimental domain. The contradictory outcomes for native and isotope labelled GEs indicated formation of native GEs during the bromination reaction. The magnitude of GE formation was influenced by the parameter settings. Consequently, strong bias on quantitative results may be expected if the isotope labelled internal standard reacts differently than native GEs. Therefore, it was necessary to explore this phenomenon in more detail. Particularly the effect of partial acylglycerols on analytical results for GEs was evaluated.

### 3.2 Influence of partial acylglycerols

To ensure a high yield in the conversion of GEs to 3-MBPD, the opening of the epoxide ring of GEs is carried out under acidic conditions. However, the acidic environment might have an impact on the trueness of the analysis, as acidic media are known to enhance formation of halogenated propanediols by the reaction of partial acylglycerols with halide ions (Ermacora & Hrncirik, 2012). Hence, the presence of partial acylglycerols in the sample matrix could lead to the artefact generation of GEs, as sodium bromide is used in excess for the conversion of GEs to 3-MPBDEs.

The optimization of critical parameters for the conversion of GEs to 3-MBPDEs in fats and oils was reported by Ermacora and Hrncirik (2013). They described the side reaction of partial acylglycerols with bromide ions. However, they suppressed this side reaction by lowering the concentration of sodium bromide in the mixture to levels that would be stoichiometrically insufficient to halogenate the potentially higher level of partial acylglycerols in oils.

**Fig. 3:**
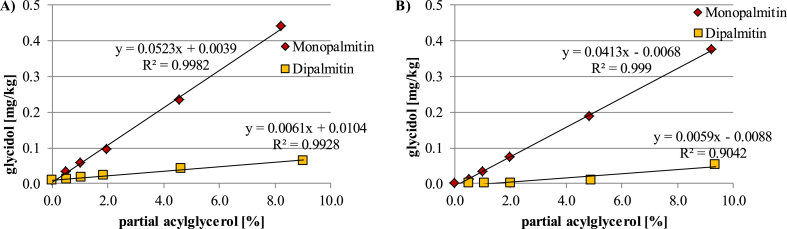
Influence of partial acylglycerol spiked into a blank oil (A) and solvent (B) on glycidol contents determined by the indirect analysis method.

The extension of this method, optimized for oils, to other food matrices could provide similar problems regarding trueness. The influence of monoacylglycerols (MAGs, represented by monopalmitin) and diacylglycerols (DAGs, represented by 1,2-dipalmitin) was tested in spiking experiments on both a blank oil matrix and in solvent (without any other acylglycerol). In both cases (with/without matrix) a linear relationships between the mono- and dipalmitin contents and formed 3-MBPD content were found. The rate of 3-MBPDE formation from monopalmitin was around ten times higher compared to dipalmitin (Fig. 3). The highest tested level of monopalmitin (about 8% w/w) in oil resulted in a GE content, expressed as glycidol, of 0.44 mg/kg compared to 0.06 mg/kg when the same oil was spiked with 9% (w/w) 1,2-dipalmitin.

The presence of MAGs and DAGs may be expected also in a variety of foods. In general, partial acylglycerols are present in edible oils and fats due to hydrolysis of triacylglycerols (TAGs) or incomplete TAG biosynthesis. MAGs are usually present in much smaller quantities than DAGs (Firestone, 2005). Hydrolytic reactions occur in oil as well as in the outer layers of food during frying; the extent depends on the frying temperature and water content (Wanasundara & Shahidi, 1998). Another source of partial acylglycerols are emulsifiers. MAGs and DAGs of fatty acids (E 471) are the most important group of food emulsifiers, accounting for about 70% of the world production (Moonen & Bas, 2004). They are widely used for optimal product formulation and processing in a variety of foods, such as baked goods (bread, cakes, crisps), margarines, mayonnaise, spreads and ice cream, (McClements, 2008, Moonen and Bas, 2004). However, significant variability of partial acylglycerol contents in food, more importantly MAGs, may be expected depending of food product and producer.

A selected group of food sample, listed in Table 3, was tested for the content of partial acylglycerols, particularly 1,2-DAGs, 1,3-DAGs and MAGs, in order to identify test items for the further evaluation of the influence of sample composition on artefact formation during the bromination step. Partial acylglycerols were determined by HPLC/ELSD. Although HPLC/ELSD is not as sensitive as mass spectrometry, it has the advantage that the detector response within a lipid class is independent of the fatty acid composition of the acylglycerols, allowing calibration with a simple mixture of reference substances. The instrument was calibrated with a diluted certified mixed standard solution in 2,2,4-trimethylpentane of 1-monoolein, 1,2-diolein and 1,3-diolein at 10–500 μg/mL. The detector parameters evaporator tube temperature (35–45 °C) and nebulizer gas flow (N_2_, 1.2–2.0 ml/min) were optimized prior to measurement of test samples. Logarithmic regression was applied for all lipid classes studied (R^2^ > 0.99), due to the non-linear detector response. Samples were further diluted in case the acylglycerol signals were outside the calibrated range. The contents of partial acylglycerols measured in the tested food samples are summarised in Table 3. As can be seen, the MAG content of many samples did not exceed 1% of the extracted fat. However, three of the samples from the food category fine bakery ware contained higher levels, between 2.0 and 12.7% MAGs.

Comparing the glycidol contents of the test samples with high MAG levels with the glycidol contents determined in blank oil fortified with MAG (Fig. 3) indicated also for certain cereal based foods a high correlation of GE content with MAG levels. For example, 2% monopalmitin spiked into blank oil resulted in a glycidol content of 0.10 mg/kg. This value is similar to that of the brioche sample (Table 3, sample 03, glycidol content of 0.12 mg/kg fat), which contained 2% of MAGs in the extracted fat. The same observation was made for sample 02 (5% MAGs, glycidol content 0.39 mg/kg fat), which was close to the glycidol content determined in the blank oil spiked with 5% MAG. The elevated amount of glycidol (0.29 mg/kg fat) in the chips sample with 0.8% MAG could be attributed to carry-over from the frying oil (Dingel & Matissek, 2015).

The contents of DAGs were in all test samples below 5%. Considering the results of spiking experiments with dipalmitin (Fig. 3), a significant increase of glycidol contents caused by artefact formation from DAGs is not expected, as a spiking level of 5% 1,2-dipalmitin in oil resulted in measured glycidol contents of 0.04 mg/kg only.

### 3.3 Comparison of GE levels determined by the indirect and direct method of analysis

The findings described above demanded an assessment of GE levels in food by a method that omits chemical transformation of the analytes. The determination of GEs by LC-MS/MS (direct method) as published by Dubois et al. (2011) for edible oil was considered most suitable for this purpose. Triaclyglycerols (TAGs) were removed from the fat extracted from test samples by GPC, prior to the measurement by LC-MS/MS. Six GEs were included in the assay (GE-L, GE-P, GE-S, GE-O, GE-Li and GE-Ln), assuming that the fatty acid composition of GEs correlate with the fatty acids of food samples (Dubois et al., 2011). Hence the most abundant fatty acids would be C16:0 and C18:0, C18:1 and C18:2 (Enig et al., 1983, Scrimgeour, 2005). The contribution of other minor fatty acids to total amount of GEs was considered low and not significant for the purpose of the exercise.

As reported also by Dubois et al. (2011), the selection of MRM transition was difficult due to the lack of selective fragment ions. Hence, the most abundant (but less selective) transitions were used for quantification. More specific fragments of either protonated fatty acids [R-COOH_2_]^+^ (GE-L, GE-P, GE-P-d_5_, GE-S) or acylium ions of fatty acids (GE-O, GE-Li, GE-Ln) were applied as qualifiers. Details are provided in Table 2. The performance of the method was assessed by spiking experiments of olive oil with the six GEs at two spiking levels (0.050 and 0.500 mg/kg). Recovery values were within the range of 92% and 103%. Relative repeatability, determined as standard deviation of three independent measurements, was better than 4% (Table 2). Potential method bias was assessed by analysing a quality control sample of soybean oil with content values derived from an interlaboratory comparison exercise. The limit of quantification was for each GE lower than 0.050 mg/kg (corresponding to glycidol equivalents 0.011–0.014 mg/kg depending on fatty acid chain), which was assumed sufficient for the purpose of comparison of results with results obtained by the indirect analysis method.

**Fig. 4:**
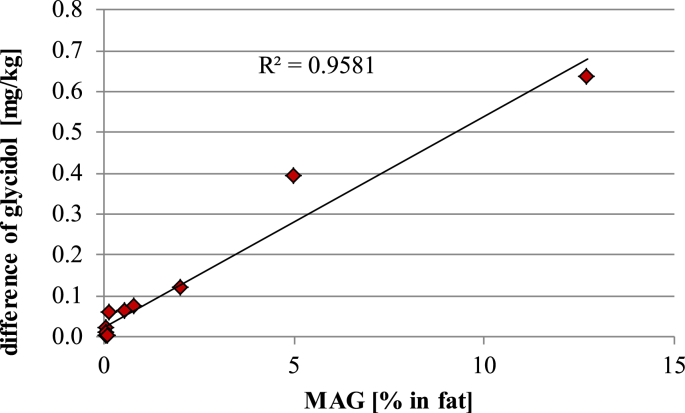
Correlation between the difference of glycidol levels determined by both the indirect GC-MS and direct LC-MS/MS analysis methods and MAGs contents in fat.

The individual GE levels determined in the tested food samples were recalculated to the glycidol equivalents and summed up. Results were compared with the results obtained by the indirect analysis method (Table 4). The amounts of glycidol determined by the two analysis methods were not significantly different for most of the samples. Statistically significant differences at 95% confidence level were found just for three samples of bakery products (Sample ID 01, 02, 03), those containing high levels of MAGs. A linear relationship was obtained when plotting the excess of glycidol formed from MAGs (difference of glycidol content measured by the direct and indirect method) and the content of MAGs determined in the extracted fat (Fig. 4). The conducted experiments confirmed a bias in the determination of GEs by the indirect method in the presence of higher amounts of MAGs in the fat fraction. However, results obtained by the two methods agreed well if the MAG content of the tested samples was low (<1% in fat).

### 3.4 SPE clean up

The direct determination of solely GEs is a valuable tool for confirmation of analysis results. However, the integration in a single method allowing the simultaneous determination of GE and 2-/3-MCPDE contents of food on the market would be more favourable for monitoring and control purposes. For making the described indirect analytical method suitable for the determination of GEs, the influence of MAGs on results of GEs has to be eliminated, or at least controlled. This can be accomplished only by introducing an additional sample clean up step. Separation according to size, as it was used for the elimination of TAGs from the fat fraction, is not possible for MAGs due to molecular weights similar to GEs. It can be achieved by solid phase extraction (SPE) on aminopropyl cartridges (NH_2_), which were used in the past for the separation of lipid classes (Vaghela & Kilara, 1995) and also successfully applied for the separation of mono- and diesters of 3-MCPD from fat (Seefelder et al., 2008). A mixture of *n*-hexane and ethyl acetate (85:15, v/v) was used as eluent. The sample intake (100 mg of the extracted fat) was kept as in the original procedure to avoid a decrease of method sensitivity. The eluent elutes the target analytes (MCPDEs and GE) together with TAGs and DAGs from the SPE cartridge, while MAGs remain on the sorbent. The elution volume was optimized by collecting 10 mL fractions up to 30 mL of eluent and analysing each fraction for MCPDE and GE contents. Experiments for method optimization were performed in triplicate using both, a soybean oil QC sample contaminated with higher amounts of MCPD mono- and diesters and GEs, but without MAGs and sample 02 (see Table 3) spiked with a low amount of MCPDEs and GEs (0.5 mg/kg) with a natural content of 5% MAGs in the fat fraction. The collected fractions were examined for the presence of MAGs by HPLC/ELSD to confirm completeness of retention of MAGs on the SPE column (Supplementary Fig. 1). All target analytes eluted within the first fraction of 10 mL eluent, MAGs were not detected in any fraction. The absolute recovery after SPE clean-up was evaluated for both samples by comparing the difference between analyte contents obtained by adding the isotope labelled internal standard either before or after SPE clean-up. The analyte recovery after SPE was between 96%–97% for 3-MCPDEs, 95%–101% for 2-MCPDEs and 84%–97% for GEs. Table 5 presents results of analysis obtained by the original and modified indirect method for both the native and spiked sample 02 and the soybean oil QC sample. Slight, but insignificant differences were observed for the measured MCPD contents, whereas statistically significant differences (95% confidence level) were found for the glycidol content in sample 02. GEs were not detectable after SPE in the native sample 02, whereas the original analysis method resulted in 0.21 mg/kg glycidol equivalents. The glycidol equivalents of the spiked sample 02 were reduced by the additional SPE to a content level equivalent to the spiking level. The determination of GEs in the soybean oil was hardly affected by the SPE (relative decrease of 4%). Analytical results for 2-MCPDEs and 3-MCPDEs were not significantly influenced by the SPE clean up.

**Fig. 5:**
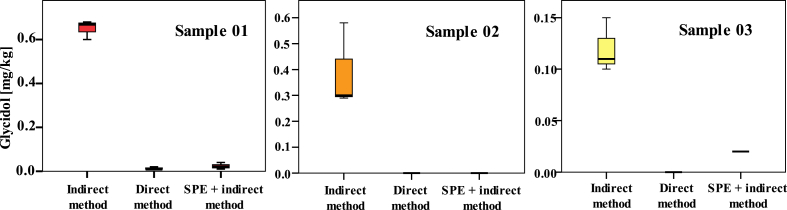
Difference in glycidol content measured by indirect GC-MS method, direct LC-MS/MS method and indirect GC-MS method after SPE clean-up.

Food samples were analysed by the indirect method including SPE clean-up. The results for GEs (expressed as glycidol equivalents), presented in Table 4, were of equal magnitude as those determined by the direct analysis method. Statistically significant differences of glycidol contents were found between the original indirect and modified indirect analysis method for samples with higher content of MAGs (sample 01, 02, 03; Fig. 5). Levels of 2-/3-MCPDEs did not differ between the original indirect method and modified indirect method with SPE.

Implementation of the SPE step also reduced to a certain degree matrix effects. Unusual variability of absolute responses of isotopically labelled internal standard GE-O-d_5_ was observed for different food matrices, whereas the responses of labelled standards of MCPDs were more constant. By including SPE, the absolute responses of labelled GE increased (for some samples up to two times) although the variability between samples was not fully eliminated.

**Fig. 6:**
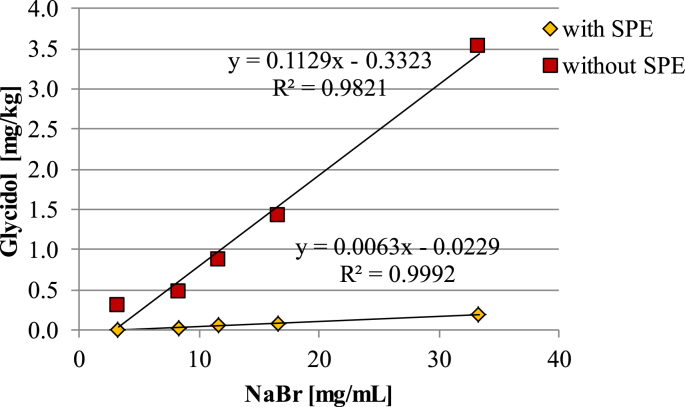
Influence of increasing amounts of sodium bromide (concentration in acidic solution) on glycidol levels measured in sample 02 by the indirect GC-MS methods after and without SPE clean up.

The effect of increasing NaBr concentrations (up to ten times the normal level) on the formation of GE was evaluated in sample 02, which contained 5% MAGs in the fat phase. A linear dependency of glycidol on the concentration of sodium bromide was found for both procedures but levels of glycidol measured by the indirect method without SPE pre-treatment were significantly higher, reaching 3.54 mg/kg for the highest sodium bromide amount compared to 0.19 mg/kg when applying SPE sample clean-up (Fig. 6).

Removing of MAGs from the fat prior to the GE conversion together with a limited amount of NaBr in the reaction mixture (30 μl of a 3.3 mg/mL solution) was found to be appropriate to ensure the results of GE obtained by indirect method are not bias.

### 3.5 Derivatization procedure

PBA is the most common derivatization agent since the methodology for MCPD has been developed. This compound is specifically reacting with diols to form volatile derivatives of MCPD or MBPD, thereby greatly improving method sensitivity and specificity. Two strategies for derivatization with PBA were so far described. One is based on reaction of the target compounds with PBA in an aqueous medium (Divinova et al., 2004, Ermacora and Hrncirik, 2013), in which PBA is introduced as a solution in acetone/water, while the second option takes place in organic solvent (Kuhlmann, 2011, Samaras et al., 2016) for which PBA is dissolved in diethyl ether. The latter was proposed to minimize the amount of PBA applied in the reaction as some research groups reported interferences and instrument stability issues caused by the introduction of high amounts of PBA into the system. The drawback of derivatization in organic solvent is the required transfer of the target compounds from an aqueous solution into an organic solution (repeated extraction with ethyl acetate) prior to derivatization. The amount of the major interferent and consumer of the derivatization reagent, glycerol is drastically reduced in this step as glycerol is just partly soluble in ethyl acetate. Similarly, as MCPDs are highly polar compounds, their extraction efficiency is also rather low.

The derivatization in organic and aqueous solvent was compared with increasing amounts of applied PBA. The absolute response of labelled and native analytes increased with increasing amount of PBA in aqueous solution. However, elevated amounts of PBA in organic solvent did not have an influence on the absolute responses of the targeted analytes even at high PBA concentrations but the responses were about 50% lower compared to derivatization in aqueous solution as specified in AOCS protocol (AOCS, 2013a). The most likely reason for this is the limited extraction efficiency of MCPDs and MBPD into organic solvent. No remarkable differences in chromatographic background were observed when comparing these two derivatization procedures, but the derivatization in an organic medium might have a positive effect on the instrument stability in long-term use.

The main pitfall experienced when using an organic solution of PBA was its solubility in diethyl ether. Three different batches of PBA obtained from one producer and one PBA purchased from another producer were compared. Approximately 0.2 g of each PBA batch were dissolved in three different solvents or mixtures of solvents and the amount of dissolved PBA was calculated. All tested PBA standards were completely dissolved in acetone/water mixture, but not all in diethyl ether (Supplementary Fig. 2). The structure and colour of the neat compound differed from batch to batch. The MS response of the targeted compounds derivatized by the different batches of PBA dissolved in diethyl ether varied (Supplementary Fig. 3) in a pattern similar to the solubility of PBA in that solvent. NMR characterisation of PBA dissolved in CDCl_3_ revealed that the commercially available PBA formulations differed with respect to their water content. One group contained a higher amount of residual water than other resulting in different solubility in diethyl ether, which consequently affects method sensitivity.

## 4 Conclusions

The standardised analytical method for determination of MCDP and glycidyl ester was critically evaluated. The conversion of GEs to MBPDEs in acidic medium was identified as the most critical step of the analytical procedure. It was demonstrated that the content of partial acylglycerols, in particular MAGs, has a negative impact on method accuracy. MAGs react with bromide in the same manner as GEs and form MBPDEs, causing thereby positively biased results. The content of the MAGs in food is critical for trueness, in particular if MAGs are used as additives in complex food such as bakery ware or generated during frying. The observed effects of MAGs on artefact formation of GEs by the indirect analysis method were confirmed by the independent direct determination of GEs by LC-MS/MS. An additional clean-up step by SPE was proposed for removing MAGs from fat prior to the conversion of GEs. This additional clean-up was applied to selected foods with an elevated MAG content, the obtained data were in agreement with result of direct analysis of GEs. The application of the additional clean-up is required at least for the analysis of food samples containing significant levels of MAGs, whereas for samples which did not contain MAGs this is not necessary.

Additionally, two different approaches for derivatization of the analytes with PBA were compared. The derivatization in both aqueous and organic media provided comparable results. In the choice of derivatization approach, the lower sensitivity and higher burden in sample manipulation of the derivatization in organic medium has to be weighed against the higher derivatization reagent consumption and associated higher potential for interferences and instrument stability issues of the derivatization in aqueous medium. Attention has to be given, especially in derivatization in organic medium, to the physical-chemical properties of the derivatization reagent, as different solubility was found for products from different batches commercialised by a particular supplier and sold under the same product number.

## Figures and Tables

**Table 1:** Detection parameters for indirect determination of MCPDEs and GEs by GC/MS.

Analyte^a^	Retention time [min]	Quantifier ion Q_1_ [m/z]	Qualifier ion Q_2_ [m/z]	Relative ion intensities^b^ [%]
3-MCPD	16.64	**147**	196	22 ± 4
2-MCPD	17.36	**196**	198	33 ± 5
3-MBPD	18.89	240	**147**	18 ± 3
3-MCPD-d_5_	16.55	**150**	201	22 ± 4
2-MCPD-d_5_	17.26	**201**	203	33 ± 5
3-MBPD-d_5_	18.80	245	**150**	18 ± 3

a Analytes are the PBA derivatives of 3-MCPD, 2-MCPD, 3-MBPD and their stable isotope labelled analogues.

b Relative intensities are expressed as a percentage of the base peak (highlighted in bold).

**Table 2:** Transition reactions and specific MRM conditions for the direct determination of GEs by LC-MS/MS, including method recovery and relative repeatability.

Analyte	Retention time [min]	Precursor ion (M+H)^+^	Product ion^a^	CE [V]^b^	CXP [V]^c^	DP [V]^d^	Recovery^e^ [%]	RSD_r_^e^ [%]
G-laurate	3.38	257.12	Q: 57.0	41	14	26	92.3; 94.9	0.4; 1.6
			C: 95.1	21	12	26		
			C: 201.0	27	12	31		
G-palmitate	7.33	313.16	Q: 57.0	43	8	46	103.3; 100.3	1.3; 1.2
			C: 70.9	46	12	46		
			C: 257.0	21	14	21		
G-steareate	11.64	341.05	Q: 57.0	53	8	36	95.8; 97.7	0.5; 1.1
			C: 85.1	25	10	36		
			C: 285.0	23	16	36		
G-oleate	8.06	338.98	Q: 55.0	63	8	36	93.6; 99.7	3.5; 1.3
			C: 69.0	49	8	36		
			C: 265.0	21	18	36		
G-linoleate	6.05	337.08	Q: 67.0	57	8	31	99.2; 103.0	2.6; 1.4
			C: 80.9	25	12	31		
			C: 263.0	19	20	26		
G-linolenate	4.79	335.03	Q: 67.0	49	8	26	98.4; 101.4	3.1; 1.5
			C: 80.9	23	4	26		
			C: 261.0	19	24	26		
G-palmitate-d_5_	7.24	318.19	Q: 57.0	47	14	21	–	–
			C: 239.0	23	16	31		
			C: 258.0	23	16	46		

a Q is the transition used for quantification, C is the transition used for confirmation.

b CE is the collision energy.

c Is the collision cell exit potential.

d Is the declustering potential.

e Results of the lower and higher spiking experiments.

**Table 3:** Fat characterisation and amount of MCPD and glycidol measured by the indirect analysis method in fat extracted from food test samples.

Sample ID	Sample description	Fat^a^ [%]	Sum DAGs [% in fat]	MAGs [% in fat]	3-MCPD [mg/kg fat]		2-MCPD [mg/kg fat]		Glycidol [mg/kg fat]	
					Average	Stdev	Average	Stdev	Average	Stdev
01	Bread	0.5	2.3	12.7	0.12	0.01	0.03	0.01	0.65	0.04
02	Roll	3.4	2.5	5.0	0.13	0.04	0.03	0.01	0.39	0.16
03	Brioche	12.1	1.6	2.0	0.11	0.03	0.03	0.01	0.12	0.03
04	Cookies	24.9	4.1	nd	1.24	0.02	0.68	0.01	0.92	0.03
05	Waffels	21.4	4.1	0.1	1.64	0.02	0.80	0.01	0.78	0.01
06	Puff pastry	33.8	4.4	0.2	1.99	0.08	0.85	0.04	1.25	0.05
07	Cornflakes	0.5	0.7	0.6	0.08	0.03	0.03	0.01	0.06	0.04
08	Popped rice	0.5	0.2	nd	0.04	0.01	<0.02	–	0.05	0.02
09	Chips	28.4	2.9	0.8	3.57	0.04	1.70	0.01	0.29	0.01
10	Smoked fish	11.3	nd	nd	0.03	0.01	nd	–	0.07	0.03
11	Fried fish	7.8	3.6	0.1	0.96	0.01	0.44	0.01	0.13	0.01
12	Bacon	18.0	1.2	0.1	nd	–	nd	–	nd	–
13	Palm oil	–	5.9	0.1	2.88	0.04	1.66	0.01	0.73	0.05
14	Heated palm oil	–	6.3	0.1	2.47	0.06	1.54	0.05	2.29	0.09
15	QC - Soybean oil	–	3.1	nd	2.72	0.13	0.12	0.01	4.39	0.04

a Share of extracted fat fraction on mass of test portion.

**Table 4:** Glycidol contents [mg/kg extracted fat] of selected test samples determined by different analysis methods.

Sample ID	Sample description	Direct method		Indirect method		Indirect method incl. SPE	
		Average	Stdev	Average	Stdev	Average	Stdev
01	Bread	0.02	–	0.65	0.04	0.02	0.01
02	Roll	nd	–	0.39	0.16	nd	–
03	Brioche	nd	–	0.12	0.03	0.02	0.01
04	Cookies	0.96	0.02	0.92	0.03	0.91	0.02
05	Waffels	0.86	0.03	0.78	0.01	0.82	0.01
06	Puff pastry	1.19	0.06	1.25	0.05	1.24	0.06
07	Cornflakes	nd	–	0.06	0.04	nd	–
08	Popped rice	0.02	0.01	0.05	0.02	0.04	0.01
09	Chips	0.22	0.01	0.29	0.01	0.22	0.01
10	Smoked fish	nd	–	0.07	0.03	<0.02	–
11	Fried fish	0.14	0.01	0.13	0.01	0.11	0.01
12	Bacon	nd	–	nd	–	nd	–
13	Palm oil	0.72	0.02	0.73	0.05	0.74	0.03
14	Heated palm oil	2.43	0.07	2.29	0.09	2.12	0.03
15	QC - Soybean oil	4.27	0.06	4.39	0.04	4.22	0.02

**Table 5:** Comparison of results [mg/kg extracted fat] measured by indirect analysis method with and without additional SPE clean-up.

Sample	Sample pre-treatment	3-MCPD		2-MCPD		Glycidol	
		Average	Stdev	Average	Stdev	Average	Stdev
02 - Roll	SPE	0.09	0.01	0.03	0.01	nd	–
	–	0.11	0.01	0.03	0.01	0.21	0.11
02 - Roll + spike	SPE	0.65	0.01	0.53	0.01	0.50	0.01
	–	0.64	0.03	0.49	0.05	0.63	0.02
QC - Soybean oil	SPE	2.72	0.03	0.10	0.01	4.19	0.01
	–	2.74	0.02	0.11	0.01	4.37	0.01
