# Supplementary material for: Assessment of critical steps of a GC/MS based indirect analytical method for the determination of fatty acid esters of monochloropropanediols (MCPDEs) and of glycidol (GEs)
Source: Food Control. 2017 Jul;77:65–75. doi: 10.1016/j.foodcont.2017.01.024 (PMC5344965; doi:10.1016/j.foodcont.2017.01.024)
Supplement: Supplementary file 1 [file mmc1.pdf]

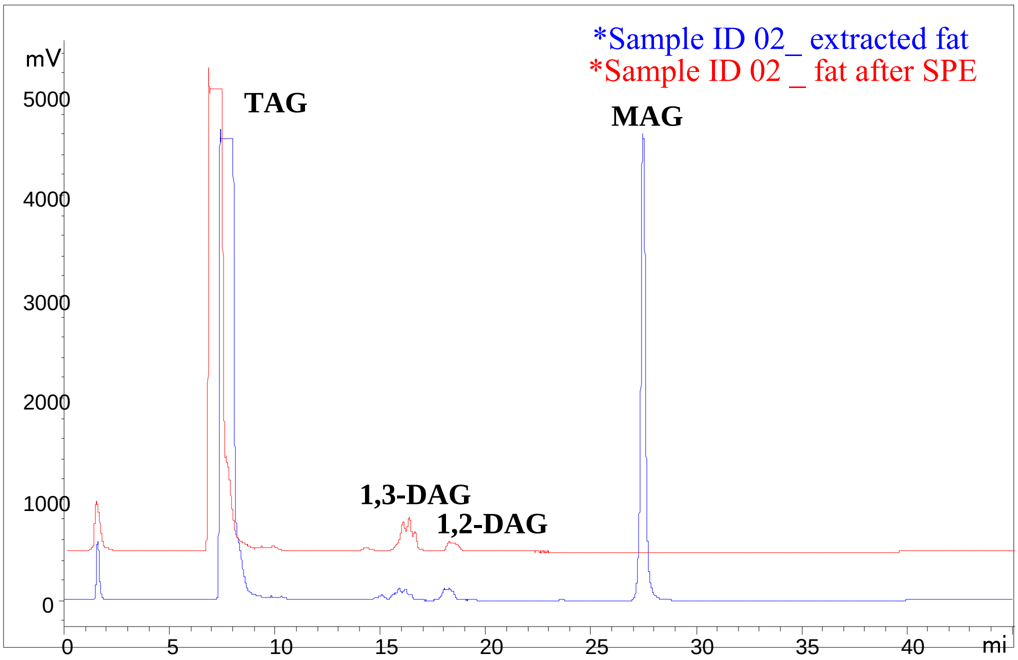

Supplementary fig. 1 Acylglycerols composition of fat sample 02 (blue line) and fat sample 02 after clean-up by SPE (red line) analysed by HPLC/ELSD

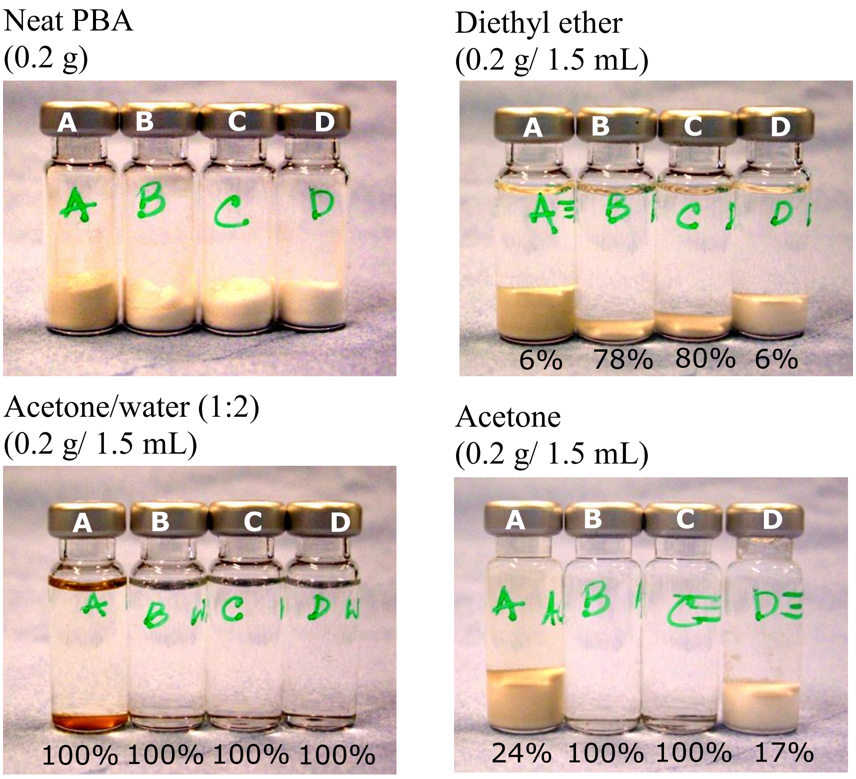

Supplementary fig. 2 Solubility of commercial batches of PBA in different solvents (A,B,C = different batches of same producer sold under same product number, D = different producer)

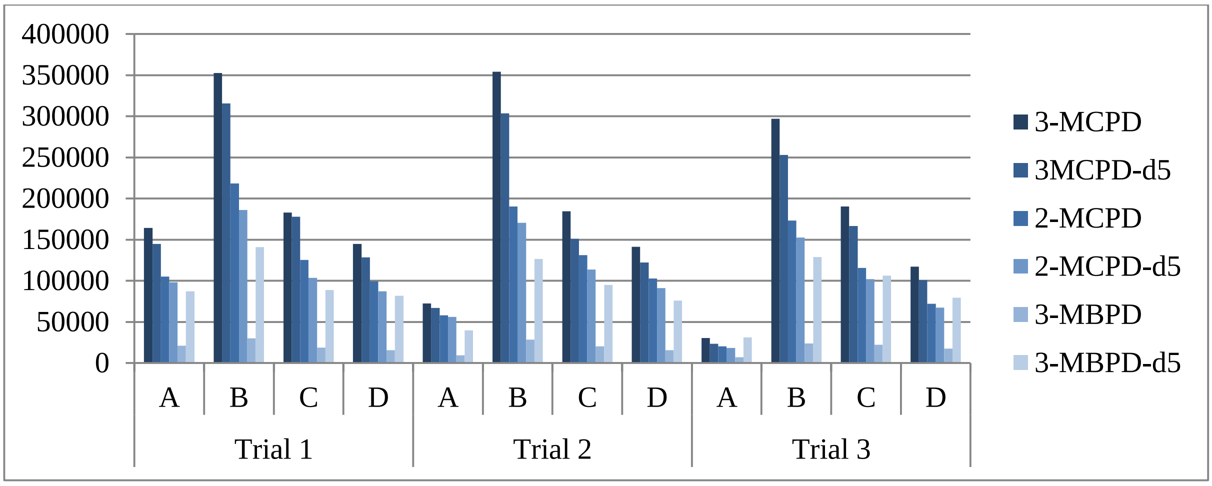

Supplementary fig. 3 Influence of different PBA reagents on the response of target compounds after derivatization in organic medium (neat PBA dissolved in diethyl ether - A,B,C = different batches of same producer, D = different producer)
